# Modeling the innate inflammatory cGAS/STING pathway: sexually dimorphic effects on microglia and cognition in obesity and prediabetes

**DOI:** 10.3389/fncel.2023.1167688

**Published:** 2023-05-03

**Authors:** Sarah E. Elzinga, Emily J. Koubek, John M. Hayes, A. Carter, Faye E. Mendelson, Ian Webber-Davis, Stephen I. Lentz, Eva L. Feldman

**Affiliations:** ^1^Department of Neurology, University of Michigan, Ann Arbor, MI, United States; ^2^Division of Metabolism, Endocrinology, and Diabetes, Department of Internal Medicine, University of Michigan, Ann Arbor, MI, United States

**Keywords:** cGAS/STING pathway, cognitive impairment, high-fat diet (HFD), obesity, inflammation, microglia

## Abstract

**Introduction:**

The prevalence of obesity, prediabetes, and diabetes continues to grow worldwide. These metabolic dysfunctions predispose individuals to neurodegenerative diseases and cognitive impairment, including dementias such as Alzheimer’s disease and Alzheimer’s disease related dementias (AD/ADRD). The innate inflammatory cGAS/STING pathway plays a pivotal role in metabolic dysfunction and is an emerging target of interest in multiple neurodegenerative diseases, including AD/ADRD. Therefore, our goal was to establish a murine model to specifically target the cGAS/STING pathway to study obesity- and prediabetes-induced cognitive impairment.

**Methods:**

We performed two pilot studies in cGAS knockout (cGAS-/-) male and female mice designed to characterize basic metabolic and inflammatory phenotypes and examine the impact of high-fat diet (HFD) on metabolic, inflammatory, and cognitive parameters.

**Results:**

cGAS-/- mice displayed normal metabolic profiles and retained the ability to respond to inflammatory stimuli, as indicated by an increase in plasma inflammatory cytokine production in response to lipopolysaccharide injection. HFD feeding caused expected increases in body weight and decreases in glucose tolerance, although onset was accelerated in females versus males. While HFD did not increase plasma or hippocampal inflammatory cytokine production, it did alter microglial morphology to a state indicative of activation, particularly in female cGAS-/- mice. However, HFD negatively impacted cognitive outcomes in male, but not female animals.

**Discussion:**

Collectively, these results suggest that cGAS-/- mice display sexually dimorphic responses to HFD, possibly based on differences in microglial morphology and cognition.

## 1. Introduction

The global burden of obesity, diabetes, and prediabetes continues to increase (Saeedi et al., 2019; Sun et al., 2022). Obesity rates have grown to pandemic proportions with over 1 billion people affected worldwide (Saklayen, 2018). In parallel, the global diabetes prevalence in 2021 topped 530 million and is estimated to grow to over 780 million by 2,045. This increase is concerning as accumulating evidence indicates that obesity, prediabetes, and diabetes predispose individuals to central nervous system (CNS) complications, including cognitive impairment and dementias such as Alzheimer’s disease and Alzheimer’s disease related dementias (AD/ADRD) (Geijselaers et al., 2017; O’Brien et al., 2017; Biessels and Despa, 2018; Henn et al., 2022b).

It is well established that obesity and metabolic dysfunction, such as prediabetes and diabetes, promote chronic low grade systemic inflammation (Luft et al., 2013; Esser et al., 2014). This inflammation extends to the brain, where high-fat diet (HFD) feeding causes an inflammatory phenotype in hippocampal microglia, the main immune cells of the CNS (Nakandakari et al., 2019; Butler et al., 2020; Elzinga et al., 2022) along with cognitive impairment (Morrison et al., 2010; Boitard et al., 2014; Sims-Robinson et al., 2016; Henn et al., 2022a). Age-related cognitive impairment and AD/ADRD are also associated with chronic low-grade system inflammation with increased reactivity/self-reactivity (De Martinis et al., 2005; Freund et al., 2010). Previous studies investigating the development of AD/ADRD during aging report that microglia function evolves over time from a protective to a chronic inflammatory phenotype, which contributes to neurodegeneration (Heneka et al., 2015; Sarlus and Heneka, 2017). Thus, chronic inflammation and immune system dysregulation may be the mechanistic link between metabolic dysfunction and cognitive impairment. However, the inflammatory mechanisms driving cognitive impairment in individuals with obesity, diabetes, or prediabetes are unknown.

While HFD-induced mouse models have provided important insight into the relationship between obesity, metabolic dysfunction, and cognitive impairment, additional relevant translational animal models are needed to further delineate the underlying inflammatory mechanisms that might contribute to this relationship. The innate inflammatory cGAS/STING (cyclic GMP-AMP/stimulator of interferon genes) pathway is an emerging target of interest in both metabolic dysfunction and cognitive impairment. The cGAS/STING pathway is highly expressed in microglia and participates in immune surveillance by sensing intracellular double-stranded DNA (dsDNA) (Chen et al., 2016; Lama et al., 2019), including endogenous dsDNA, such as mitochondrial DNAs, which can localize to the cytosol due to cellular stress (White et al., 2014; Bai et al., 2017).

In the periphery, HFD feeding increases STING expression in the liver and activates cGAS/STING signaling in adipocytes through release of mitochondrial DNAs to the cytosol (Bai et al., 2017; Qiao et al., 2018), triggering an inflammatory response (Bai et al., 2017; Mao et al., 2017). In the CNS, excessive or chronic cGAS/STING activation in microglia leads to inflammation and neurodegeneration (Paul et al., 2021; Tanaka and Takahashi, 2021) and is implicated in multiple neurodegenerative diseases, such as ataxia telangiectasia, Parkinson’s disease, amyotrophic lateral sclerosis, and Alzheimer’s disease (Sliter et al., 2018; Yu et al., 2020; Jin et al., 2021; Paul et al., 2021). Additionally, our recent work shows dysregulation of hippocampal cGAS/STING during early obesity and prediabetes and suggests a critical role for microglia in the process (Elzinga et al., 2022). Thus, cGAS/STING may be a mechanistic “bridge” between metabolic dysfunction and cognitive impairment, making murine models that specifically target cGAS/STING of great interest in understanding this connection.

Prior studies examining the role of cGAS/STING signaling in HFD-induced obesity and metabolic dysfunction have primarily used STING, and not cGAS, knockout mouse models. Indeed, STING knockout protects mice against HFD-induced obesity, inflammation, insulin resistance, apoptosis, and glucose intolerance (Bai et al., 2017; Mao et al., 2017; Qiao et al., 2018). However, while cGAS is primarily activated by dsDNA to promote a type 1 interferon inflammatory response, STING can act independent of cGAS, and has additional roles in cellular programs such as regulation of autophagy, induction of lysosomal cell death, or metabolic regulation (Abe and Barber, 2014; Gaidt et al., 2017; Liu et al., 2019; Vila et al., 2022). Therefore, our goal was to create and characterize a model to better explore the role of cGAS/STING specific signaling in the CNS during obesity and prediabetes. To do so, we performed two pilot studies in cGAS knockout mice. The first was used to characterize the metabolic and inflammatory phenotype of these animals while on maintenance diet. The second was used to understand the role of cGAS/STING under clinically relevant conditions of obesity and prediabetes and characterize the resulting metabolic, inflammatory, and cognitive responses. As cGAS/STING-induced inflammation is thought to contribute to cognitive impairment, we hypothesized that cGAS knockout mice would have reduced microglial activation, lower inflammation, and thus be partially protected from HFD-induced cognitive decline.

We observed that cGAS knockout (cGAS-/-) male and female mice have similar metabolic profiles and retain their ability to respond to inflammatory stimuli while on maintenance diet. HFD feeding caused systemic changes in metabolic responses, with increased body weight and plasma insulin and impaired glucose tolerance compared to standard diet (SD) fed animals. Interestingly, onset of this metabolic phenotype was delayed in male but accelerated in female cGAS-/- mice. cGAS knockout protected both sexes from HFD-induced increases in inflammatory cytokine concentrations in plasma and hippocampal tissue. However, HFD did alter microglial morphology to a more amoeboid shape, indicative of activation, but to a greater extent in female versus male cGAS-/- mice. Interestingly, HFD fed male cGAS-/- mice displayed cognitive impairment relative to SD fed males, whereas HFD females were not significantly different from their SD controls. Collectively, these findings suggest that male and female cGAS-/- mice exhibit similar metabolic and inflammatory responses but display sexually dimorphic microglial morphology and cognition responses to HFD feeding. The results in these exploratory pilot studies indicate that cGAS-/- mice are an adequate working model to examine response to HFD. They also provide the basis for additional larger comprehensive studies exploring the molecular mechanisms underlying the role of cGAS/STING in obesity and metabolic dysfunction-driven cognitive impairment.

## 2. Materials and methods

### 2.1. Experimental animals and study design

**FIGURE 1:**
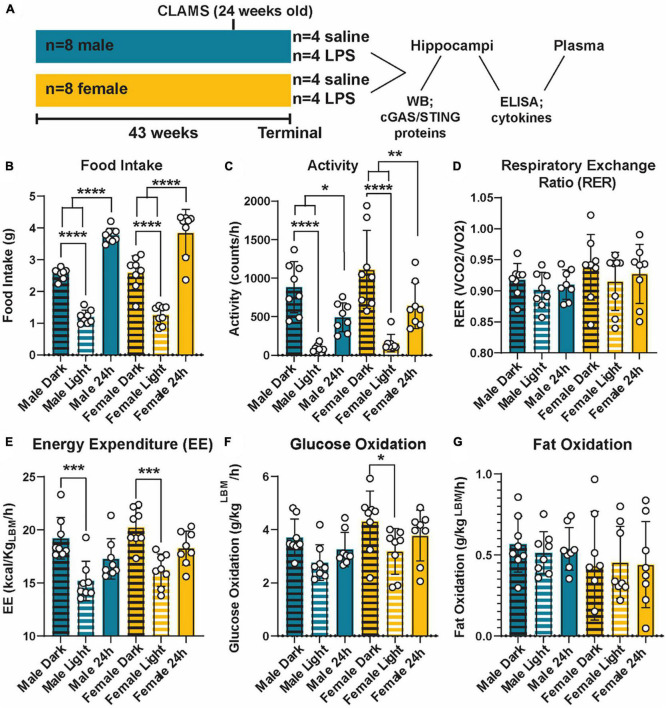
Metabolic phenotyping of cGAS-/- mice. Experimental timeline **(A)**. Food intake **(B)**, activity **(C)**, respiratory exchange ratio [RER; **(D)**], energy expenditure [EE; **(E)**], glucose oxidation **(F)**, and fat oxidation **(G)** in 5.5 mo male and female cGAS-/- mice during light:dark cycle. *n* = 8 mice/group, *p < 0.05, **p < 0.01, ***p < 0.001, and ****p < 0.0001, two-way ANOVA followed by Tukey’s *post-hoc* testing for multiple comparisons. Data are presented as the mean ± s.e.m.

Data were obtained from two cohorts of mice. Cohort 1 was used to determine the impact of cGAS knockout on basic metabolic and inflammatory responses (Figure 1A). Cohort 1 was comprised of male (*n* = 8) and female (*n* = 8) B6(C)-cGAStm1d(EUCOMM)Hmgu (strain #026554; Jackson Laboratory, Bar Harbor, ME, USA) mice kept on a maintenance diet (5LOD; 5.7% calories from fat; LabDiets, Saint Paul, MN, USA). At study termination, mice were 43 weeks old and were randomly administered either a single intraperitoneal injection of lipopolysaccharide (LPS) or saline as a control. LPS (catalog #tlrl-3pelps, Invivogen, San Diego, CA, USA, *n* = 4 mice/group/sex) was given at a dose of 500 μg LPS/kg body weight in a total volume of 5 mL/kg body weight. Saline was also administered at 5 mL/kg body weight, *n* = 4 mice/group/sex. Immediately after injection, mice were fasted, euthanized, and samples were collected.

Cohort 2 was used to explore metabolic, inflammatory, and cognitive responses to HFD (Figure 4A). Cohort 2 consisted of male (*n* = 8) and female (*n* = 10) B6(C)-cGAStm1d(EUCOMM)Hmgu/J (cGAS-/-; strain #026554; Jackson Laboratory) mice. At 5 weeks of age, mice were randomly assigned to either a standard diet (SD; *n* = 4 males and *n* = 5 females; 10% calories from fat; catalog #D12450J; Research Diets, New Brunswick, NJ, USA) or HFD (*n* = 4 males and *n* = 5 females; 60% calories from fat; catalog #D12492; Research Diets). After 25 weeks on diet, terminal phenotyping and sample collection were performed.

For both cohorts, mice were fasted for 4 h prior to euthanasia via intraperitoneal injection of 150 mg/kg pentobarbital (Fatal-Plus, Vortech Pharmaceuticals, Dearborn, MI, USA). Before tissue removal, blood was taken from the vena cava and animals were perfused with phosphate buffered saline. Hemi-brains were collected for analysis of microglial morphology. Hippocampal tissue was used to quantify cGAS/STING protein expression by Western blot. Hippocampal tissue and plasma were analyzed for inflammatory cytokine concentrations by ELISA.

All mice were housed in a pathogen-free room (20 ± 2°C, 12 h light/dark cycle) at the University of Michigan Unit for Laboratory Animal Medicine with no more than five littermates per cage for SD animals and no more than 3 littermates per cage for HFD animals. Water and food were provided *ad libitum*, as well as a minimum of one enrichment item (nestlet and/or enviropak). Mice were monitored daily by University of Michigan veterinary staff. All protocols and procedures were approved by the University of Michigan Committee on the Use and Care of Animals, and were in compliance with university guidelines, state and federal regulations, and the standards of the “Guide for the Care and Use of Laboratory Animals.”

### 2.2. Metabolic phenotyping

A Comprehensive Laboratory Monitoring Systems (CLAMS; Columbus Instruments, Columbus, OH, USA) unit was used as an indirect calorimeter to measure food intake and activity, respiratory exchange ratio, energy expenditure, glucose oxidation, and fat oxidation. CLAMS analysis was performed at the University of Michigan Metabolic, Physiological, and Behavioral Phenotyping Core.

For SD versus HFD comparison, body weight was measured weekly. Terminal plasma insulin was measured by University of Michigan Metabolic, Physiological, and Behavioral Phenotyping Core. Glucose tolerance testing (GTT) was performed 1, 4, 8, 12, 16, 20, and 24 weeks after diet initiation as previously described (Hinder et al., 2017; O’Brien et al., 2018). Briefly, animals were fasted for 4 h. Blood glucose measurements were taken at scheduled intervals over 2 h post-administration of a 10% D-glucose bolus. D-glucose was given via intraperitoneal injection at 1 g/kg body weight. Blood glucose was measured from a tail blood sample with an AlphaTrak Glucometer (Abbot Laboratories, Chicago, IL, USA) and appropriate glucose strips (Zoetis, Parsippany, NJ, USA).

### 2.3. Microglial morphology

As previously described (York et al., 2018; Elzinga et al., 2022), microglial morphology was analyzed in the hilus, molecular layer, and CA1 regions of the hippocampus. Briefly, dissected hemi-brains were fixed for 48 h in 4% paraformaldehyde. Hemi-brains were exposed to a sucrose gradient (10, 20, and 30% sucrose for 24 h each) before embedding in OCT and frozen at −80°C. Brains were sectioned (50 μm) and stained as floating tissue sections in 6-well plates. Tissue was stained for ionized calcium binding adaptor molecule 1 (IBA-1; rabbit anti-Iba1, 1:1000; catalog #019-19741, Wako Chemicals, Richmond, VA, USA), secondary antibody (anti-rabbit Alexa-fluor Plus 594, 1:2000; catalog #A32740; Invitrogen, Waltham, MA, USA), and Hoechst nuclear stain. Prolong Gold (Invitrogen) was used to mount stained sections and Z-stack images (30 μm) were obtained with a Leica Stellaris 8 Falcon Confocal Microscope (Leica Microsystems, Wetzlar, Germany). Imaris Software (Oxford Instruments, Abingdon, United Kingdom) was used to process images and microglial morphology was analyzed by open microscopy environment TIF files using a modified 3DMorph script in Matlab (Mathworks, Natick, MA, USA), as previously described (York et al., 2018).

### 2.4. ELISA and western blotting

As previous (Elzinga et al., 2022), blood and hippocampal tissue were collected 4 h following LPS stimulation or 25 weeks after diet initiation for inflammatory cytokine analysis by ELISA and for cGAS/STING pathway protein expression via Western Blot. In preparation for ELISA and Western blot, hippocampal tissue was homogenized in Pierce™ RIPA buffer (Thermo Fisher Scientific, Waltham, MA, USA) plus proteinase inhibitor, sonicated, and centrifuged for 30 min at 4°C at 13,300 rpm (Kim et al., 2015; Kim et al., 2019). TNF-α, IL-6, IL-10, and MCP-1 were quantified via ELISA by the University of Michigan Rogel Cancer Center Immunology Core (Ann Arbor, MI, USA). Hippocampal cytokine concentrations (pg/mL) were normalized (i.e., divided by) to total protein concentrations (μg/μL) for a final tissue cytokine concentration expressed as pg/mg of protein.

For Western blotting, samples were run on SDS-PAGE and transferred to nitrocellulose membranes. Membranes were blocked in 5% bovine serum albumin (BSA) in tris-buffered saline (TBS) with 0.01% Tween-20 (TBS-T) for 2 h. Membranes were incubated overnight at 4°C with the following primary antibodies from Cell Signaling Technologies (Danvers, MA, USA) in TBS-T: cGAS (catalog #31659S; 1:1000), STING (catalog #50494S; 1:1000), pIRF3 (S396; catalog #4947S; 1:500), total IRF3 (catalog #4302S; 1:500), and NFκB (catalog #8242; 1:1000). Tubulin (catalog #ab6160; 1:20000; Abcam, Cambridge, MA, USA) or GAPDH was used as a loading control (catalog #sc-31915; 1:1000; Santa Cruz, Dallas, TX, USA). The next day, membranes were incubated for 1.5 h at room temperature with anti-rabbit (catalog #7074) or anti-rat (catalog #7077S) secondary antibodies from Cell Signaling Technologies. Protein signals were visualized using Clarity Max (Bio-Rad, Hercules, CA, USA). Images were captured using a ChemiDoc (Bio-Rad) and analyzed using Image Lab software (Bio-Rad).

### 2.5. Puzzle box

As described previously, a modified version of the puzzle box task was performed to assess possible changes in cognition due to HFD (Williams et al., 2020; Elzinga et al., 2022). For each task, the animal must exit the light area of the box and enter the dark area. Puzzle box testing was carried after 25 weeks on SD or HFD over a period of 3 days. Over the course of the first 2 days of testing, 4 single tasks were repeated for a total of 3 replicates. Single tasks were as follows; (1) open door with nothing blocking the exit to the dark area of the box, (2) movable nestlet (cotton square) attached at the top inside of the door to the exit, (3) Envirodri (crinkled paper) bunched up outside the door to the exit, and (4) tunnel (4-way PVC pipe). Single tasks were then combined into a “complex” task, with the nestlet attached at the top inside of the door, tunnel in front of the door, and the Envirodri in the three arms of the tunnel not in front of the door. The complex task was performed once at the end of day 2 and again 24 h later on day 3. The time it took the mouse to “escape” the light area and enter the dark area of the box was recorded for each task. A cut-off of 5 min was given for the mouse to perform each task. Inability to escape the light area within 5 min resulted in removal of the mouse from the box and a recorded time of 5 min.

### 2.6. Statistical analysis

Statistical analyses were performed using either Prism (version 9; GraphPad Software, La Jolla, CA) or SAS 9.4 software (SAS Institute, Cary, NC). Statistical tests used were two-way analysis of variance (ANOVA) or two-way repeated measures ANOVA followed by Tukey’s testing for multiple comparison correction. Analysis of microglial morphology was performed using SAS 9.4 (SAS Institute, Cary, NC) using the Proc Mixed function with the number of microglia per region set as a random effect. Statistical significance was defined as *p* < 0.05. Results are presented as mean ± standard error of the mean (SEM).

## 3. Results

### 3.1. cGAS-/- mice display normal metabolic profiles

Given the interconnection between metabolic and innate inflammatory pathways (Brestoff and Artis, 2015; Buck et al., 2017), we first examined the impact of cGAS knockout on metabolic parameters across sex (Figure 1A). cGAS-/- males and females mice displayed similar metabolic profiles (Figure 1), which were roughly comparable to what is reported in the literature (Supplementary Table 1) (Marvyn et al., 2016; Soofi et al., 2017; Franczyk et al., 2021). Additionally, both sexes had an expected higher food intake and activity during dark periods compared to light (Figures 1B, C). This corresponded to a higher energy expenditure during those periods (Figure 1E). We did not observe differences in respiratory exchange ratios, glucose oxidation, or fat oxidation between light/dark periods or across sexes (Figures 1D, F, G), with the exception of a small decrease in glucose oxidation in the light vs. dark period in the females that was not observed in the males (Figure 1F). Therefore, we concluded that cGAS knockout male and female animals maintain similar and expected metabolic responses.

### 3.2. cGAS-/- mice respond to inflammatory stimuli

**FIGURE 2:**
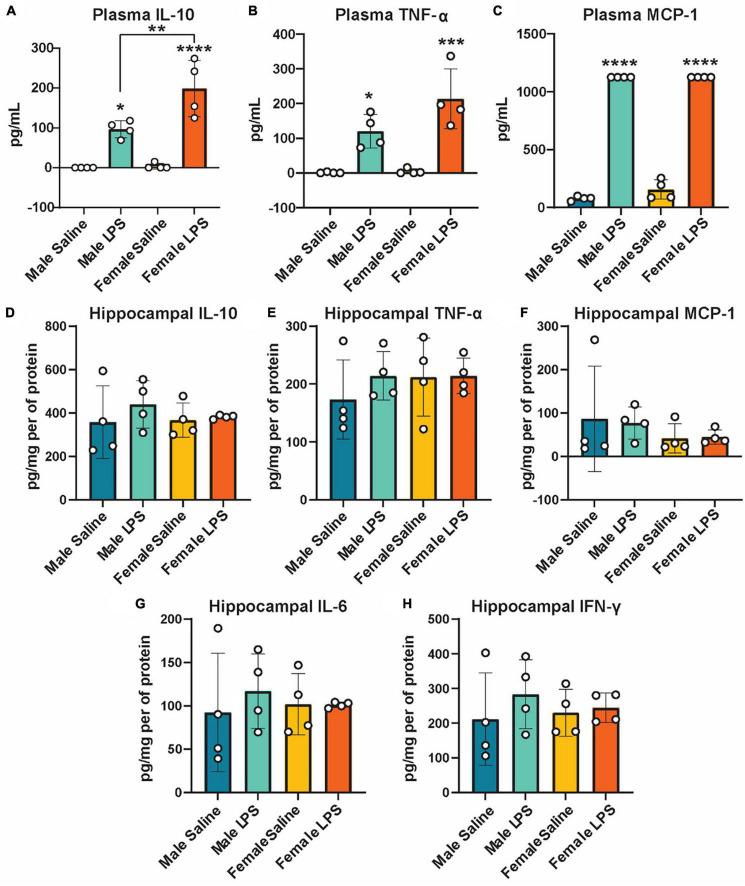
Plasma and hippocampal inflammatory cytokines following LPS stimulation as measured by ELISA. Plasma IL-10 **(A)**, TNF-α **(B)**, and MCP-1 **(C)** and hippocampal IL-10 **(D)**, TNF- α **(E)**, MCP-1 **(F)**, IL-6 **(G)**, and IFN-γ **(H)** concentrations in male and female cGAS-/- mice administered either saline or lipopolysaccharide (LPS). *n* = 4 mice/group, *p < 0.05, **p < 0.01, ***p < 0.001, ****p < 0.0001, two-way ANOVA followed by Tukey’s *post-hoc* testing for multiple comparisons. Data are presented as the mean ± s.e.m.

**FIGURE 3:**
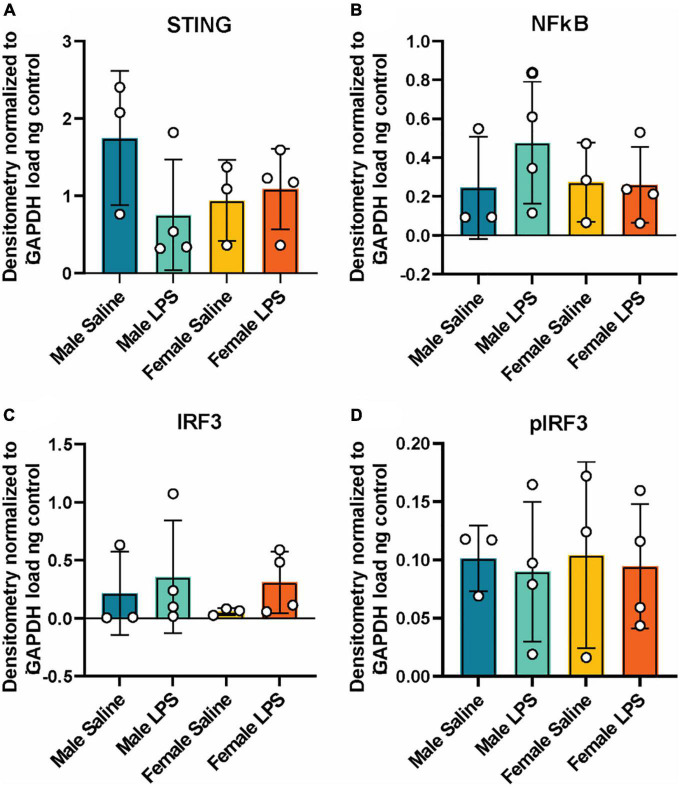
Hippocampal cGAS/STING protein expression following LPS stimulation. Expression in male and female cGAS-/- mice administered either saline or lipopolysaccharide (LPS). Data represented as STING **(A)**, NFκB **(B)**, total IRF3 **(C)** and pIRF3 **(D)** relative protein expression. Protein expression quantified as average band intensity relative to GAPDH loading control. *n* = 3 mice/group for saline, *n* = 4 mice/group for LPS, two-way ANOVA followed by Tukey’s *post-hoc* testing for multiple comparisons. Data are presented as the mean ± s.e.m.

Next, to determine if cGAS-/- mice can respond to inflammatory stimuli, we administered either LPS as an immune activator or saline as a control, and measured plasma and hippocampal inflammatory cytokine concentrations. We observed a robust increase in plasma IL-10, TNF-α, and MCP-1 concentrations in both male and female cGAS-/- mice following LPS (Figures 2A–C), in line with values from other published reports (Supplementary Table 2) (Li et al., 2019; Elzinga et al., 2022). Of note, the increase in plasma IL-10 following LPS injection was greater in females compared to males (Figure 2A). However, there were no difference in hippocampal concentrations of IL-10, TNF-α, MCP-1, IL-6, or IFN-γ between stimulated and unstimulated cGAS-/- mice, regardless of sex (Figures 2D–H). This is different from previously published data (Supplementary Table 3) (Frühauf et al., 2015; Zhao et al., 2019), likely due to multiple factors, including differences in mouse strain or LPS dosing regimen. As expected, LPS stimulation did not alter hippocampal expression of STING and cGAS was not detectible in hippocampal lysate (Figure 3A; Supplementary Figure 1). Additionally, changes in hippocampal expression or phosphorylation of cGAS/STING pathway proteins, including transcription factors NFκβ (Figure 3B; Supplementary Figure 1B) or IRF3 (Figures 3C, D; Supplementary Figures 1C, D), were not observed. Overall, these results indicate that, at least in terms of peripheral inflammatory responses, cGAS-/- mice can respond to LPS stimulation.

**FIGURE 4:**
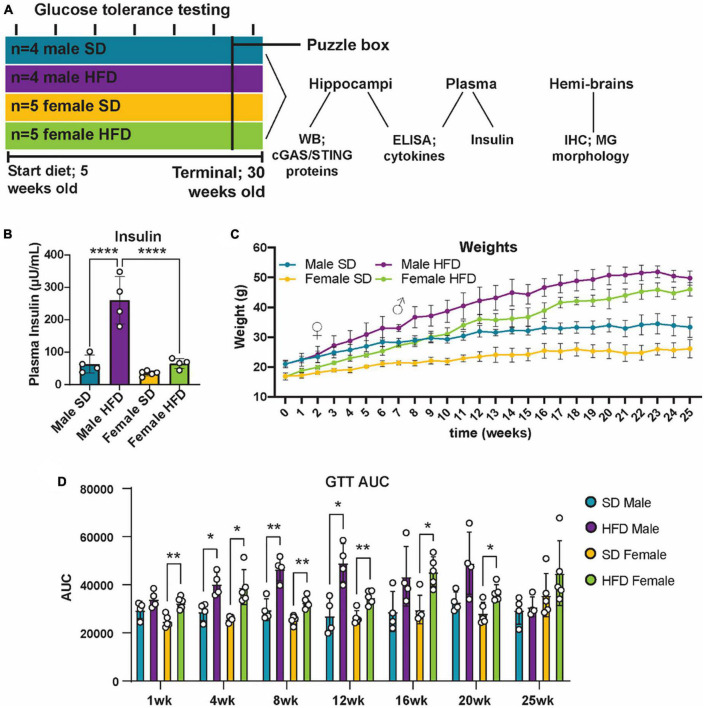
Metabolic phenotyping following high-fat diet. Experimental timeline **(A)**. Plasma insulin **(B)**, body weight **(C)**, and glucose tolerance test area under the curve [GTT AUC; **(D)**], in male and female cGAS-/- mice fed standard diet (SD) or high-fat diet (HFD) for 25 weeks. *n* = 4 male mice and *n* = 5 female mice/group; *p < 0.05, **p < 0.01, ****p < 0.0001, ♀ indicates the point at which female HFD mice weighed more than female SD mice and ♂ indicates the point at which male HFD mice weighed more than male SD mice, two-way ANOVA or two-way repeated measured ANOVA followed by Tukey’s *post-hoc* testing for multiple comparisons. Data are presented as the mean ± s.e.m.

### 3.3. High-fat diet impairs metabolic responses in cGAS-/- mice

It is well established that chronic and acute high-fat diet (HFD) feeding induces obesity and prediabetes in non-transgenic mice, and that chronic feeding promotes cognitive impairment (Sims-Robinson et al., 2016; Hinder et al., 2017; O’Brien et al., 2020; Elzinga et al., 2022). However, less is known regarding the impact of cGAS/STING specific signaling on metabolic response following HFD, and how those responses might impact cognition. To examine this, cGAS-/- mice were placed on either a SD or HFD for 25 weeks (Figure 4A). Body weight was measured weekly and both male and female cGAS-/- mice fed HFD gained weight over the course of the study (Figure 4C). Interestingly, female cGAS-/- mice fed HFD gained weight quickly and were significantly heavier than female cGAS-/- mice fed SD after only 2 weeks on diet. In contrast, male cGAS-/- mice on HFD were not heavier than their SD counterparts until after 7 weeks on diet. However, after these time points, both male and female HFD fed cGAS-/- mice were significantly heavier than their SD counterpoints for the remainder of the study. Glucose tolerance testing (GTT) was performed at week 1 and subsequently every 4 weeks beginning at week 4 (Figure 4D; Supplementary Figure 2). HFD feeding resulted in impaired glucose tolerance, regardless of sex, after only 1 week on diet (Supplementary Figure 2), although GTT area under the curve was not different after 1 week in males (Figure 4D). Impaired glucose tolerance in HFD mice persisted until week 24, at which point blood glucose levels during GTT in HFD mice were no longer significantly different from SD fed mice (Figure 4D; Supplementary Figure 2). Male cGAS-/- mice on HFD had significantly higher plasma insulin levels at study termination than male cGAS-/- mice on SD. However, plasma insulin levels of female cGAS-/- HFD mice were no different from male or female cGAS-/- mice on SD (Figure 4B), which is in line with previously published reports (Supplementary Table 4) (Elzinga et al., 2021). Taken together, these results indicate that cGAS potentially plays a sex-dependent role in metabolic response to HFD, delaying HFD-induced weight gain in male cGAS-/- animals, but accelerating it in female cGAS-/- animals.

### 3.4. High-fat diet does not alter hippocampal inflammatory profiles in cGAS-/- mice

**FIGURE 5:**
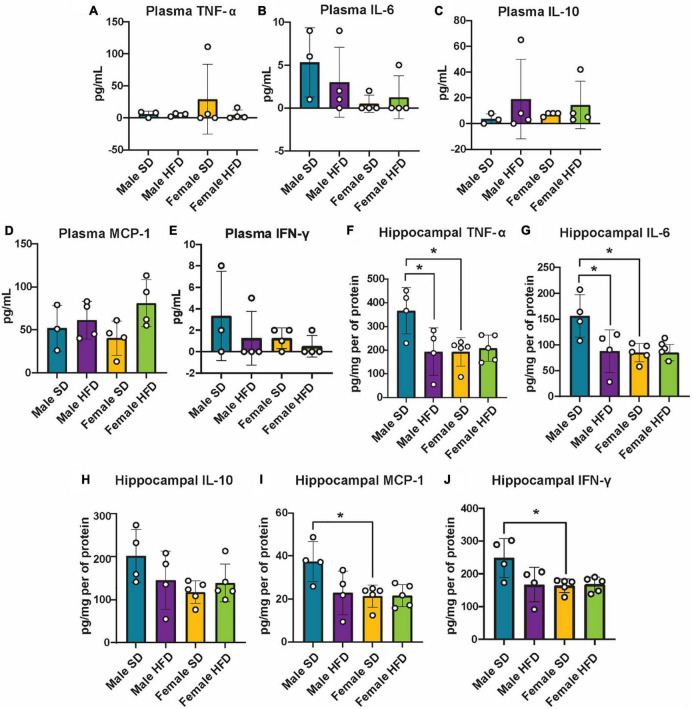
Plasma and hippocampal inflammatory markers following high-fat diet as measured by ELISA. Plasma TNF-α **(A)**, IL-6 **(B)**, IL-10 **(C)**, MCP-1 **(D)**, IFN-γ **(E)**, and hippocampal TNF-α **(F)**, IL-6 **(G)**, IL-10 **(H)**, MCP-1 **(I)**, IFN-γ **(J)** concentrations in male and female cGAS-/- mice fed a standard diet (SD) or a high-fat diet (HFD) for 25 weeks. *n* = 4 mice/group for plasma and male hippocampal samples, *n* = 5 mice/group for female hippocampal samples, *p < 0.05, two-way ANOVA followed by Tukey’s *post-hoc* testing for multiple comparisons. Data are presented as the mean ± s.e.m.

**FIGURE 6:**
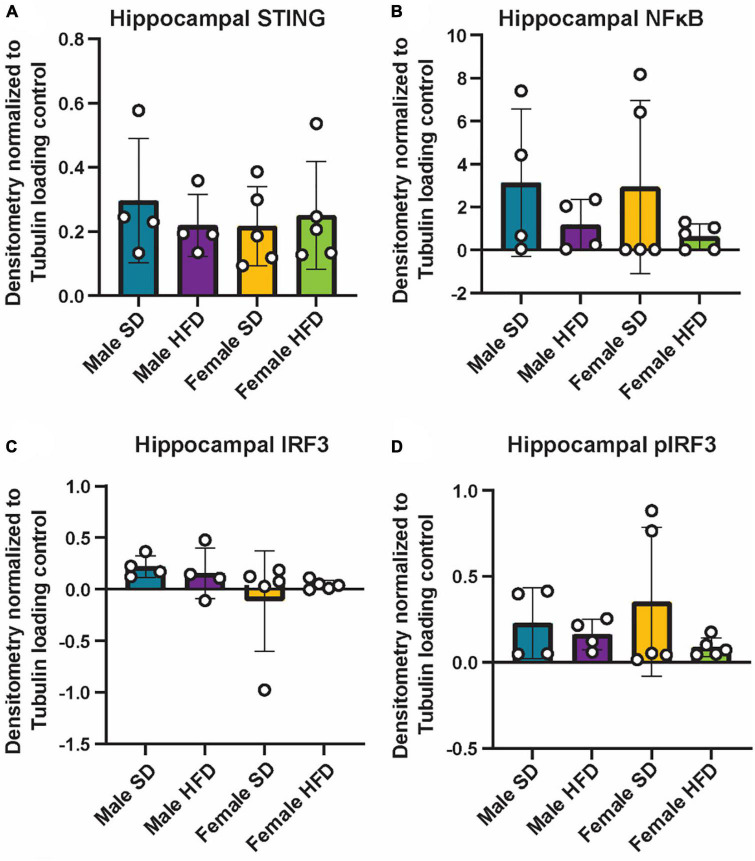
Hippocampal cGAS/STING protein expression following high-fat diet. Expression in male and female cGAS-/- mice fed standard diet (SD) or high-fat diet (HFD) for 25 weeks. Data represented as STING **(A)**, NFκB **(B)**, total IRF3 **(C)**, and pIRF3 **(D)** relative protein expression. Protein expression quantified as average band intensity relative to tubulin loading control. *n* = 4 mice/group for male cGAS-/-, *n* = 5 mice/group for female cGAS^–/–^ mice, two-way ANOVA followed by Tukey’s *post-hoc* testing for multiple comparisons. Data are presented as the mean ± s.e.m.

We and others have previously reported that chronic and acute HFD induces an inflammatory phenotype in non-transgenic C57BL/6J mice, including alterations in cGAS/STING signaling (Supplementary Table 5) (Denver et al., 2018; Elzinga et al., 2019, 2022; Henn et al., 2022a). We did not observe similar changes in circulating inflammatory cytokine concentrations in cGAS-/- mice in response to HFD feeding (Figures 5A–E; Supplementary Table 5), although an inflammatory response was present following immune challenge (LPS injection, Figures 2A–C). In parallel to peripheral cytokine levels, concentrations of brain inflammatory cytokines can also increase in response to HFD feeding in non-transgenic C57BL/6J mice (Supplementary Table 6) (Yang et al., 2019; Henn et al., 2022a). Here, this response too was blunted in the hippocampus of male cGAS-/- mice fed HFD, with lower levels of TNF-α and IL-6 when compared to cGAS-/- males fed SD (Figures 5F, G). Hippocampal IL-10 concentrations did not differ between SD and HFD mice, regardless of sex (Figure 5H). SD female cGAS-/- mice also had lower hippocampal MCP-1 and IFN-γ concentrations compared to SD male cGAS-/- mice (Figures 5I, J). As with LPS, we also confirmed that HFD does not change hippocampal protein levels of STING, NFκB, IRF3 (Figures 6A–C; Supplementary Figure 3), or phosphorylated IRF3 (Figure 6D; Supplementary Figure 3D) in either sex. Given the established pro-inflammatory effect of HFD in non-transgenic C57BL/6J mice (Denver et al., 2018; Elzinga et al., 2019, 2022), and the non-significant impact of HFD in cGAS-/- animals, these data suggest that cGAS-/- may protect animals from HFD-induced inflammatory changes.

### 3.5. High-fat diet alters microglial morphology particularly in female cGAS-/- mice

**FIGURE 7:**
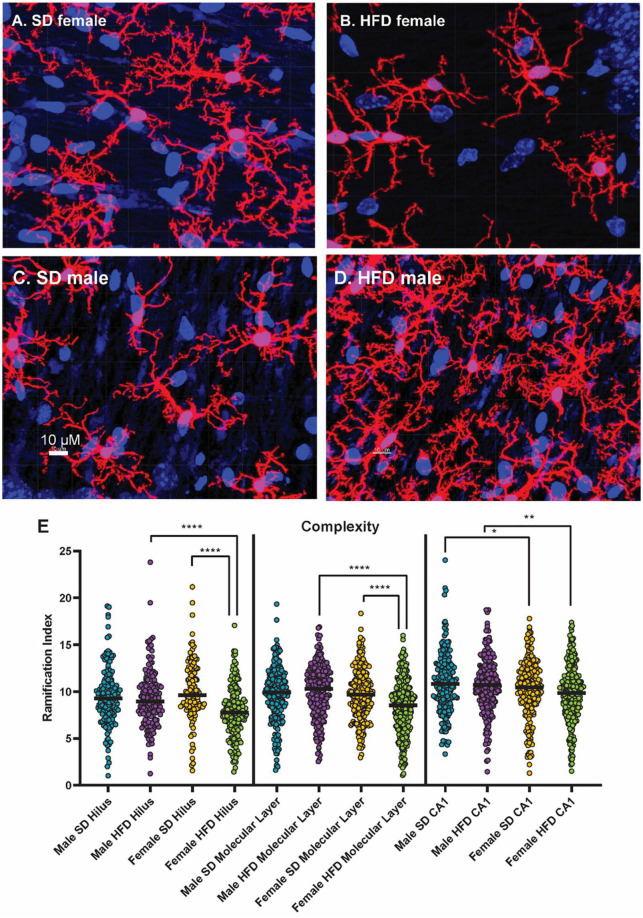
Microglial morphology following high-fat diet. Representative images of IBA-1 microglia (red stain) in female cGAS-/- mice fed standard diet [SD female, **(A)**] or high-fat diet [HFD female, **(B)**] and in male cGAS-/- mice fed standard diet [SD male, **(C)**] or high-fat diet [HFD male, **(D)**] for 25 weeks. Quantification of microglia ramification index in the hilus, molecular layer, and CA1 hippocampal regions in male and female cGAS-/- mice fed SD or HFD **(E)**. *n* = 4 SD males, *n* = 4 HFD males, *n* = 4 SD females, *n* = 5 HFD females, *p < 0.05, **p < 0.01, ****p < 0.0001, mixed model (SAS Proc Mixed) with number of cells per region set as a random effect. Data are presented as the mean ± s.e.m.

We have previously determined that acute HFD feeding activates hippocampal microglia in non-transgenic C57BL/6J male mice (Elzinga et al., 2022). In both male and female cGAS-/- mice, HFD shifted the morphology of hippocampal microglial to a more amoeboid shape indicative of activation (Figure 7; Supplementary Figures 4–6). HFD-induced morphological changes were particularly pronounced in the molecular layer and CA1 regions of the hippocampus. When quantifying these changes, we observed that HFD, regardless of sex, decreased microglial cell territory (volume within the three-dimensional perimeter of the microglia), volume (three-dimensional volume of the microglia alone), and branch length. Female cGAS-/- mice on HFD had a significantly decreased ramification index (ratio of cell territory to cell volume) of microglia in their molecular layer and hilus compared to female cGAS-/- mice on a SD. However, differences in ramification index were not observed between male SD or HFD fed cGAS-/- mice, regardless of hippocampal region. These data collectively suggest cGAS/STING signaling may impact HFD-induced activation of hippocampal microglia in a sexually dimorphic manner, where cGAS knockout may be protective in males but deleterious or without impact in females.

### 3.6. High-fat diet impairs cognitive responses in male, but not female, cGAS-/- mice

**FIGURE 8:**
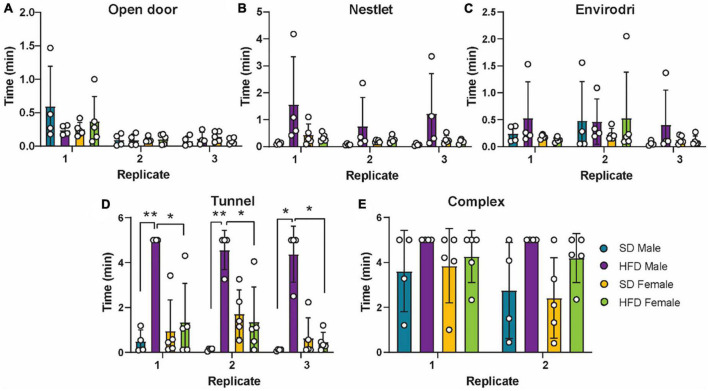
Cognition as measured by puzzle box testing. Data represented as single tasks, open door **(A)**, nestlet **(B)**, envirodri **(C)**, and tunnel **(D)**, and the combination of the single tasks into a complex task **(E)** in male and female cGAS-/- mice fed standard diet (SD) or high-fat diet (HFD) for 25 weeks. *n* = 4 mice/group for male cGAS-/- mice, *n* = 5 mice/group for female cGAS KO mice, *p < 0.05, **p < 0.01, two-way repeated measured ANOVA followed by Tukey’s *post-hoc* testing for multiple comparisons. Data are presented as the mean ± s.e.m.

Finally, we and others have shown that HFD induces cognitive impairment in non-transgenic mice (Sims-Robinson et al., 2016; Denver et al., 2018; Miranda et al., 2018). To explore the impact of cGAS-/- on HFD-induced cognitive impairment across sex, we performed puzzle box testing (Figure 8). Puzzle box testing uses a series of increasingly difficult behavioral tasks, which primarily test executive function. We observed impaired cognitive functioning in male cGAS-/- mice fed a HFD compared to male cGAS-/- mice on a SD, as evidenced by an increased latency to escape the box during the tunnel task (Figure 8D). On the other hand, we did not detect any differences in behavior between female SD and HFD-fed cGAS-/- mice for any of the tasks measured (Figure 8). These data suggest that cGAS-/- mice display different cognitive responses to HFD based on sex.

## 4. Discussion

Obesity, prediabetes, and diabetes induce peripheral and central inflammation and increase the risk of cognitive impairment. However, the precise mechanisms underlying metabolic dysfunction-induced inflammation and cognitive impairment are unknown. Therefore, additional clinically relevant mouse models of cognitive decline are needed to elucidate the biological mechanisms that underlie metabolic dysfunction, inflammation, and cognitive impairment. The dsDNA sensing cGAS/STING inflammatory pathway may potentially act as a mechanistic “bridge” between the two pathologies, making cGAS specific models particularly useful in delineating this connection. In the current study, we performed two pilot studies in a cGAS-/- mouse model to characterize the effects of HFD on metabolism, inflammation, and cognition. We hypothesized that cGAS-/- mice would have reduced microglial activation, lower inflammation, and thus be partially protected from HFD-induced cognitive decline. Our data show that metabolic profiles in cGAS-/- mice do not differ across sex and that both sexes are able produce a robust inflammatory in response to stimuli in the form of a LPS injection. When fed HFD, cGAS-/- mice gain weight and display glucose intolerance. Interestingly, while HFD did not cause increased inflammatory cytokine production in cGAS-/- mice, it does alter microglial morphology to a state that indicates microglial activation. However, this HFD-induced morphological change, was primarily observed in female cGAS-/- animals. Conversely, changes in cognition were detected in HFD-fed male, but not female, cGAS-/- mice. Overall, our findings suggest that cGAS/STING signaling may play a sex-specific role in susceptibility to HFD-induced obesity, microglial activation, and cognitive responses. They also support the future use of cGAS-/- mice for the study of HFD-induced inflammatory mechanisms that promote cognitive impairment.

We found that lack of cGAS did not impact basic metabolic phenotype across sex. Indeed, the metabolic profile of cGAS-/- mice consistent with literature regarding non-transgenic C57BL/6J mice, the recommended control (Marvyn et al., 2016; Schipper et al., 2020; Casimiro et al., 2021). Indeed, these studies report similar respiratory exchange ratios, glucose oxidation, and fat oxidation rates. Additionally, they describe a similar greater food intake, activity, and energy expenditure during the dark cycle compared to the light cycle. The findings presented here are also aligned with another study of cGAS-/- mice, which did not observe changes in body weight, glycemia, or rectal temperature between male cGAS-/- and non-transgenic mice on a normal diet (Vila et al., 2022). In this same study, STING deficient mice displayed increased food intake and glucose uptake compared to wild type mice, without changes in body weight (Vila et al., 2022). Metabolic alterations observed in the absence of STNG suggest that STING, but not cGAS, deficiency impacts metabolic parameters independently of the cGAS/STING pathway. Collectively, these results suggest that, unlike STING, cGAS deficiency may not impact the metabolic profile of mice. However, additional studies directly comparing cGAS-/- and non-transgenic mice are required to fully assess the impact of cGAS deficiency on metabolism.

We also observed that cGAS-/- animals can mount an inflammatory response to LPS stimulation. Specifically, LPS increased levels of circulating inflammatory cytokines, with a greater increase in IL-10 observed in female mice compared to males. In both cGAS and STING-deficient mice, LPS stimulation increases plasma levels of IL-6, TNF-α, and MCP-1, roughly equivalent to what is described in non-transgenic mice in the literature (Li et al., 2019; Visitchanakun et al., 2021; Elzinga et al., 2022). While LPS produced a robust increase in circulating inflammatory cytokines, we did not see any change in hippocampal cytokine concentrations. This is perhaps not surprising as protein expression of inflammatory cytokines, particularly in CNS tissue, can be highly variable. Indeed, while changes in cytokine gene expression in response to stimuli are routinely reported in the literature (Boitard et al., 2014; Wu et al., 2018; Wang et al., 2022), changes in protein expression are either not reported or not observed (Skelly et al., 2013). As expected, LPS did not alter protein expression or phosphorylation of cGAS/STING proteins, including IRF3 or NFκB, transcription factors downstream of cGAS/STING that trigger type 1 interferon production.

Consistent with literature regarding chronic HFD, cGAS-/- mice fed a HFD displayed increases in body weight and impaired glucose tolerance (Sims-Robinson et al., 2016; Hinder et al., 2017). However, by week 24, differences in glucose tolerance between SD- and HFD-fed animals were no longer observed. This is likely due to age-related metabolic changes in SD mice (Basu et al., 2003; Chia et al., 2018). While terminal GTTs showed no differences due to diet, we observed sexually dimorphic differences in terminal plasma insulin and weight gain in cGAS-/- animals fed HFD. Unlike males, females fed a HFD for 25 weeks did not develop hyperinsulinemia. This finding is consistent with other studies of sexual dimorphism in response to dietary fat (Hwang et al., 2010; Gelineau et al., 2017). Interestingly, in the current study, female cGAS-/- mice on a HFD were significantly heavier than mice on a SD after only 2 weeks of feeding. Conversely, males on HFD were not heavier compared to their SD controls until after 7 weeks on diet. This contrasts with previous studies using non-transgenic C57BL/6 mice where females display delayed weight gain in response to HFD feeding compared to males, who gain weight quickly (Yang et al., 2014; Casimiro et al., 2021; Elzinga et al., 2021; Oraha et al., 2022). However, our results align with another study that used a mouse model with a Cx3cr1 knockout to explore sex-specific differences in HFD feeding (Dorfman et al., 2017). Cx3cr1 is a highly expressed chemokine receptor on microglia which has critical homeostatic roles in the brain (Zhang et al., 2012; Sellner et al., 2016). Similar to the results presented here, female Cx3cr1 knockout mice fed a HFD displayed a marked early increase in body weight similar to their male counterparts. These results suggest that intact cGAS signaling during HFD feeding may protect female mice from weight gain but promote weight gain in males. However, how cGAS provides a sex-specific protective effect is unclear and requires additional investigation.

Release of cytosolic dsDNA in response to metabolic stress caused by a HFD or saturated fatty acid overload triggers cGAS/STING activity in the periphery (Bai et al., 2017; Mao et al., 2017; Yuan et al., 2017). HFD-induced cGAS/STING signaling promotes pro-inflammatory responses that may contribute to the pathogenesis of obesity and prediabetes/diabetes (Chen et al., 2016; Bai and Liu, 2019). We observed that, unlike with LPS, HFD feeding did not increase plasma inflammatory cytokine concentrations in cGAS deficient mice. STING knockout has similarly been shown to impact inflammatory processes, decreasing HFD-induced macrophage infiltration into adipose tissue (Mao et al., 2017). Collectively, these results suggest that cGAS signaling may play a role in HFD-induced inflammatory responses. While HFD upregulates levels of cGAS and STING in non-transgenic type animals (Bai et al., 2017), we-as expected-did not observe any changes in hippocampal protein expression of STING or the downstream signaling factors IRF3 and NFκB in knockout animals. Interestingly, male cGAS-/- mice fed a SD displayed higher hippocampal levels of TNF-α, IL-6, and MCP-1 compared to cGAS-/- SD-fed female and cGAS-/- HFD-fed male and female animals. We noted that these elevated hippocampal inflammatory cytokine concentrations were not observed in the first cohort of male mice treated with saline. This result may be an artifact of the small sample size and additional investigation with a larger cohort of mice, including non-transgenic animals, is needed to provide additional clarification.

Microglia, resident immune cells of the CNS, have high levels of cGAS/STING and produce type I interferons upon cGAS/STING activation (Olson and Miller, 2004; Jack et al., 2005; Murira and Lamarre, 2016). In non-transgenic animals, HFD induces hippocampal microglial activation with concurrent cGAS/STING activation (Nakandakari et al., 2019; Butler et al., 2020; Elzinga et al., 2022). However, these prior studies primarily used male mice to examine to the impact of HFD on microglial activation. In this study, we included both male and female cGAS-/- mice and found that HFD induced significant changes in microglial morphology indicative of activation primarily in female animals. Emerging evidence supports the existence of sexually dimorphic microglial responses to HFD or other stressors. Indeed, male and female microglia show differences in cell morphology, density, transcriptional profiles, and cellular functions (Morselli et al., 2014; Dorfman et al., 2017; Han et al., 2021). In non-transgenic C57BL6/J mice, HFD increases hypothalamic microglial activation in male, but not female, mice (Thaler et al., 2012; Valdearcos et al., 2014; Dorfman et al., 2017). However, and similar to the data presented here, others found that HFD fed CX3CR1-deficient female mice had more hypothalamic microglia and reduced microglial processes length, indicating a greater degree of microglial activation (Dorfman et al., 2017). Additionally, there is controversy within the field as to the appropriate methods to measure microglial activation. Microglial morphology and microglial ‘activation’ are a dynamic process in these cells and changes in morphology alone may not be the best proxy to measure activation (Paolicelli et al., 2022). Indeed, morphology can be influenced by multiple factors, such as age, brain location, sex, and environment. Sex-based differences in immune-related pathways, such as cGAS/STING, may contribute to sex-based differences in microglial activation. However, these differences may also be partly explained by epigenetic mechanisms, endocrine factors, and microenvironmental signals (Cheray and Joseph, 2018; Han et al., 2021). Thus, additional investigation into sex-specific microglial activation (particularly using multiple robust measures of activation), and the potential role of cGAS/STING, are required.

Involvement of cGAS/STING signaling in the periphery in metabolic dysfunction, such as obesity and prediabetes/diabetes is well-documented. However, less is known regarding the importance of the cGAS/STING pathway in the CNS under these same conditions. Recently, aberrant or sustained cGAS/STING activation has been observed in a number of neurodegenerative diseases and is thought to contribute to neuronal dysfunction via microglial-induced CNS neuroinflammation and neurodegeneration (Sliter et al., 2018; Yu et al., 2020; Jin et al., 2021; Paul et al., 2021). Thus, we would expect that cGAS deficiency would partially protect mice fed a HFD from cognitive decline. Here we found that HFD-fed male, and not HFD-fed female, cGAS-/- mice have cognitive deficits in the tunnel task compared to their SD-fed counterparts. Previous studies have shown that female mice fed a HFD display deficits in cognitive performance that are associated with obesity and glucose intolerance (Johnson et al., 2016; Salinero et al., 2020; Gannon et al., 2022). Therefore, as HFD induced early obesity, glucose intolerance, and microglial activation in female cGAS-/- mice fed HFD, we expected to observe impaired cognitive response in females. Instead, female cGAS-/- fed a HFD did not have significant differences in their puzzle box performance relative to female cGAS-/- animals fed SD, indicating that they may be protected from HFD-induced cognitive impairment. Conversely, and despite their delayed weight gain, HFD-fed male cGAS-/- animals performed worse on the tunnel task than their SD-fed counterparts. Sex differences exist in the incidence rate, age of onset, and severity of neurodegenerative diseases and may be partly due to dysfunctional microglia (Doust et al., 2021). Additionally, early development of CNS diseases is more common in males, while later development is more prevalent in females (Bilbo, 2017). However, despite known sex-based differences in microglial function and activity, there are limited studies examining sex differences in HFD-induced cognitive impairment. Therefore, as microglial activation evolves over time, the microglial activation observed in HFD females might precede cognitive deficits. Conversely, cGAS-/- may be protective against cognitive impairment in females, but not in males. Cognitive impairment is also a complex outcome that can involve multiple cell types and multiple regions of the brain. Here, we assessed cognition by Puzzle box testing, which primarily measures executive functioning and is not brain region specific (Nada et al., 2011; Williams et al., 2020). Puzzle box testing is dependent upon the animal’s anxiety in the light area of the box and motivation to escape to small dark spaces (Nada et al., 2011). Incorporating additional tasks to directly measure anxiety or motor deficits, such as open field task (Eltokhi et al., 2020), or tasks that assess cognitive impairment in specific regions of the brain, should be incorporated in future studies. Additional investigation exploring sexually dimorphic responses to HFD in regard to microglial activation and cognitive impairment, particularly including non-transgenic animals, are also required.

Our study does have some limitations. First, it was carried out as two pilot studies with the purpose of informing the basis and design of additional, more comprehensive work. As such, the number of animals used in each experiment is small. However, the results obtained are consistent with those observed in previous studies. Additionally, cGAS-/- mice were not profiled against non-transgenic mice. We are currently performing additional analyses with larger cohorts of both cGAS-/- and non-transgenic mice. Finally, the study used a mouse model with global cGAS knockout. Future studies exploring cell specific or conditional cGAS knockout, such as in microglia or hippocampus, would allow for precise delineation of the local role of cGAS/STING signaling in the CNS, without confounding effects from the periphery.

Overall, our data indicate that cGAS-/- male and female mice have similar metabolic phenotypes and retain their ability to respond to immune stimuli. HFD feeding promotes weight gain and dysregulated glucose metabolism in cGAS-/- mice, the onset of which appears to be sexually dimorphic. Interestingly, differences in microglial activation and cognitive function were also observed between sex following HFD. Specifically, HFD induced microglial activation primarily in female cGAS-/- mice, but impaired cognitive function in male, and not female, animals. The results presented in these pilot studies suggest a novel sexually dimorphic role for cGAS in HFD-induced microglial activation. They also suggest that HFD cGAS-/- mice are a viable model to investigate innate inflammatory mechanisms connecting metabolic dysfunction and cognitive impairment and provide an important foundation for additional studies.

## Data availability statement

The original contributions presented in this study are included in the article/Supplementary material, further inquiries can be directed to the corresponding author.

## Ethics statement

The animal study was reviewed and approved by the University of Michigan Committee on the Use and Care of Animals.

## Author contributions

SE and EF designed the studies. SE, JH, AC, FM, and IW-D acquired the data. SE analyzed the data. SE, EK, SL, and EF interpreted the data. SE and EK wrote the manuscript. SE, EK, and EF edited the manuscript. All authors reviewed and approved the submitted version.
