# Supplementary material for: Modeling the innate inflammatory cGAS/STING pathway: sexually dimorphic effects on microglia and cognition in obesity and prediabetes
Source: Front Cell Neurosci. 2023 May 3;17:1167688. doi: 10.3389/fncel.2023.1167688 (PMC10188944; doi:10.3389/fncel.2023.1167688)
Supplement: Supplementary file 1 [file Data_Sheet_1.DOCX]

**TITLE PAGE**

**Title:** Innate inflammatory cGAS/STING; sexually dimorphic effects on microglia and cognition in a model of obesity and prediabetes

**Authors:** Sarah E. Elzinga, Emily J. Koubek, John M. Hayes, Faye E. Mendelson, Ian Webber-Davis, Stephen I. Lentz, Eva L. Feldman

**Corresponding author:**

Eva Feldman, MD, PhD, FAAN, FANA

University of Michigan

Department of Neurology

109 Zina Pitcher Place, 5017 AAT-BSRF

Ann Arbor, MI 48109-220, USA

Phone: 734-763-7274 / Fax: 734-763-7275

E-mail: efeldman@med.umich.edu

**Supplemental Figures**

**
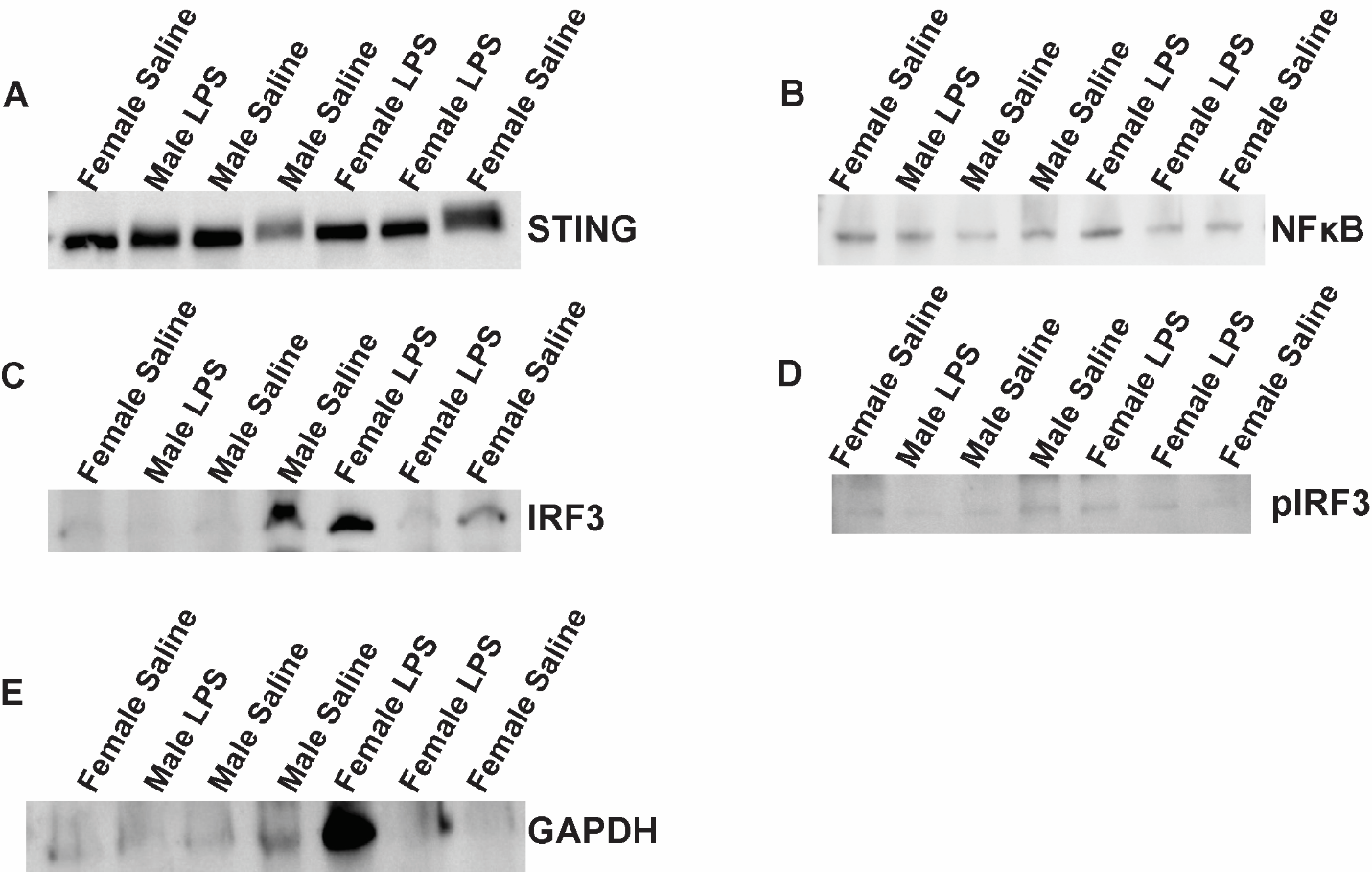
**

**Supplemental Fig 1.** Representative Western blot images of hippocampal lysates from male and female cGAS-/- mice administered either saline or lipopolysaccharide (LPS).

**
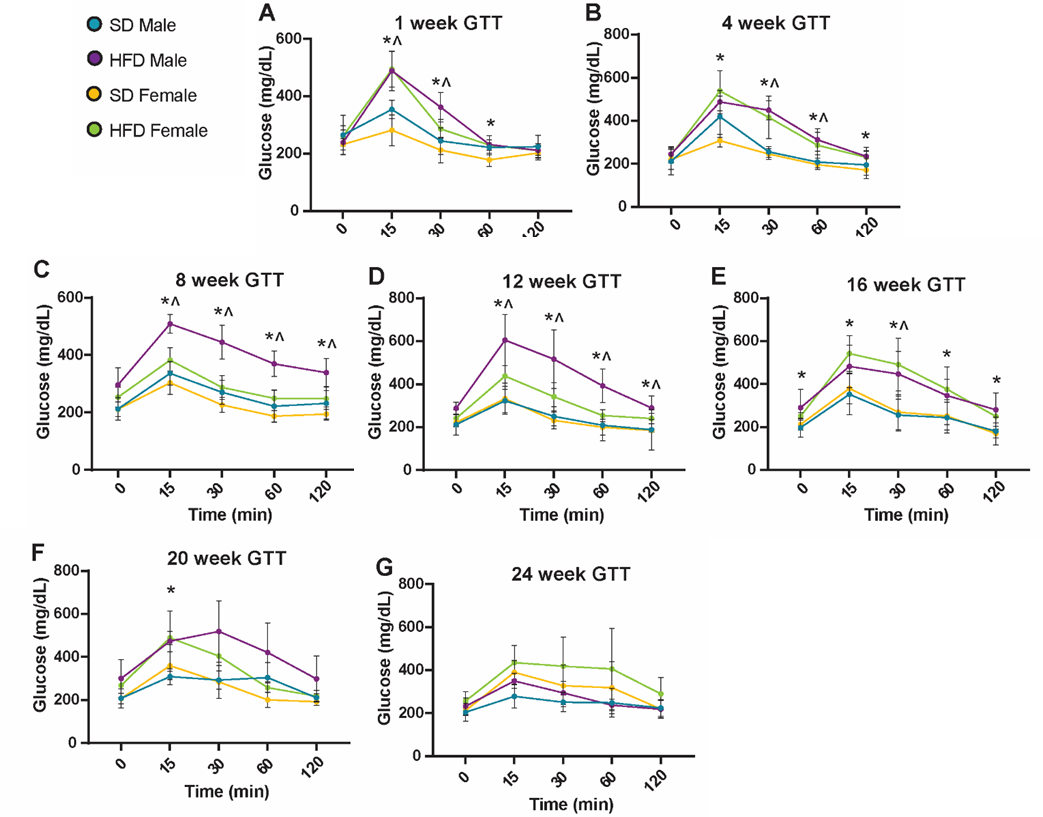
**

**Supplemental Fig 2.** Longitudinal glucose tolerance testing (GTT) in male and female cGAS-/- mice fed standard diet (SD) or high-fat diet (HFD) for 25 weeks. *n* = 4 male mice and *n* = 5 female mice/group, **p* < 0.05 for female SD vs female HFD, ^*p* < 0.05 for male SD vs male HFD; 2-way repeated measured ANOVA followed by Tukey’s post hoc testing for multiple comparisons. Data are presented as the mean ± s.e.m.


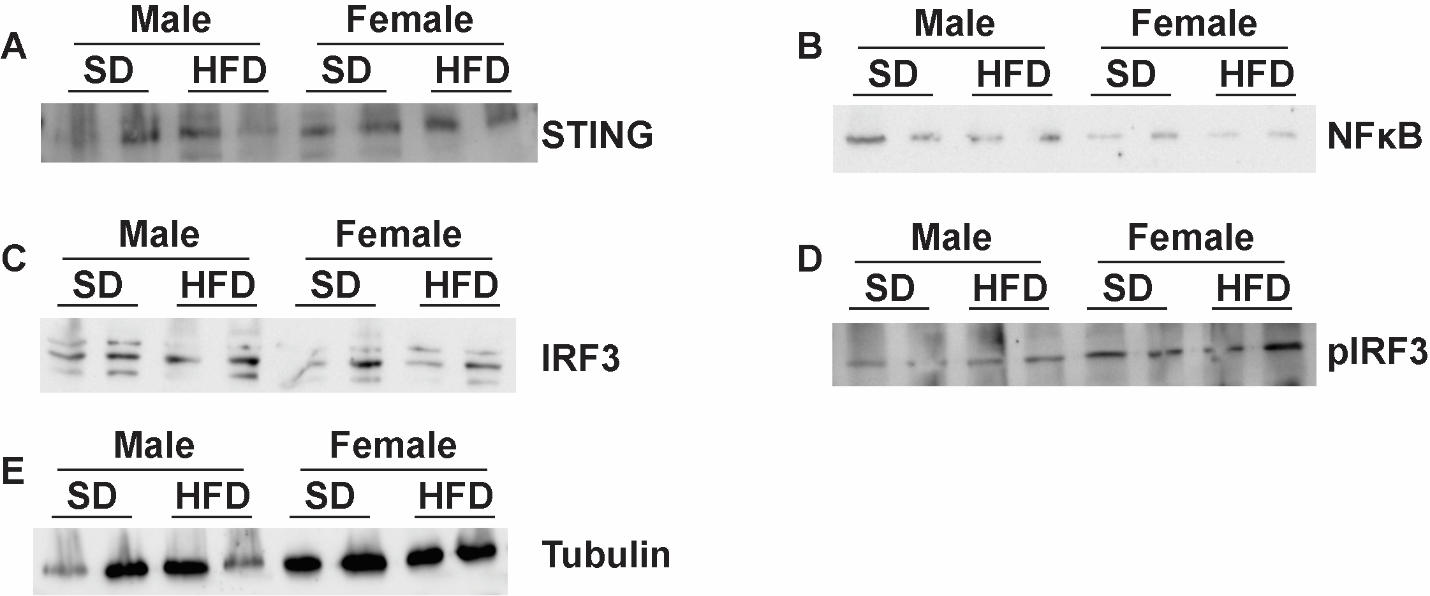


**Supplemental Fig 3.** Representative Western Blot images of hippocampal lysates from male and female cGAS-/- mice fed standard diet (SD) or high-fat diet (HFD) for 25 weeks.


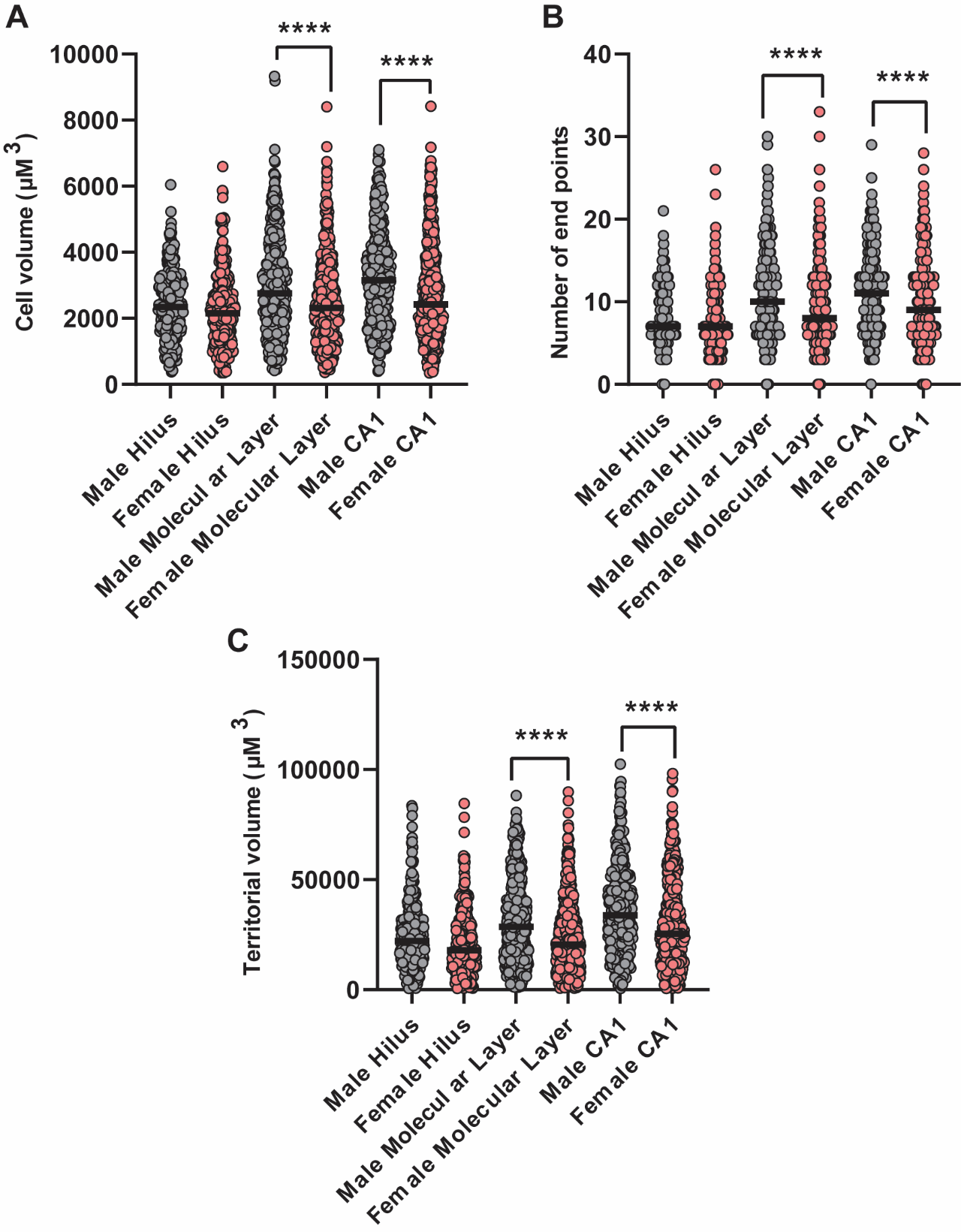


**Supplemental Fig 4.** Microglial morphology differences in male and female cGAS -/- mice per hippocampal region. Quantification of cell volume (A), number of end points (B), and territorial volume (C) in the hilus, molecular layer, and CA1 hippocampal regions. *n* = 8 males, *n* = 9 females, *****p* < 0.0001, mixed model (SAS Proc Mixed) with number of cells per region set as a random effect. Data are presented as the mean ± s.e.m.


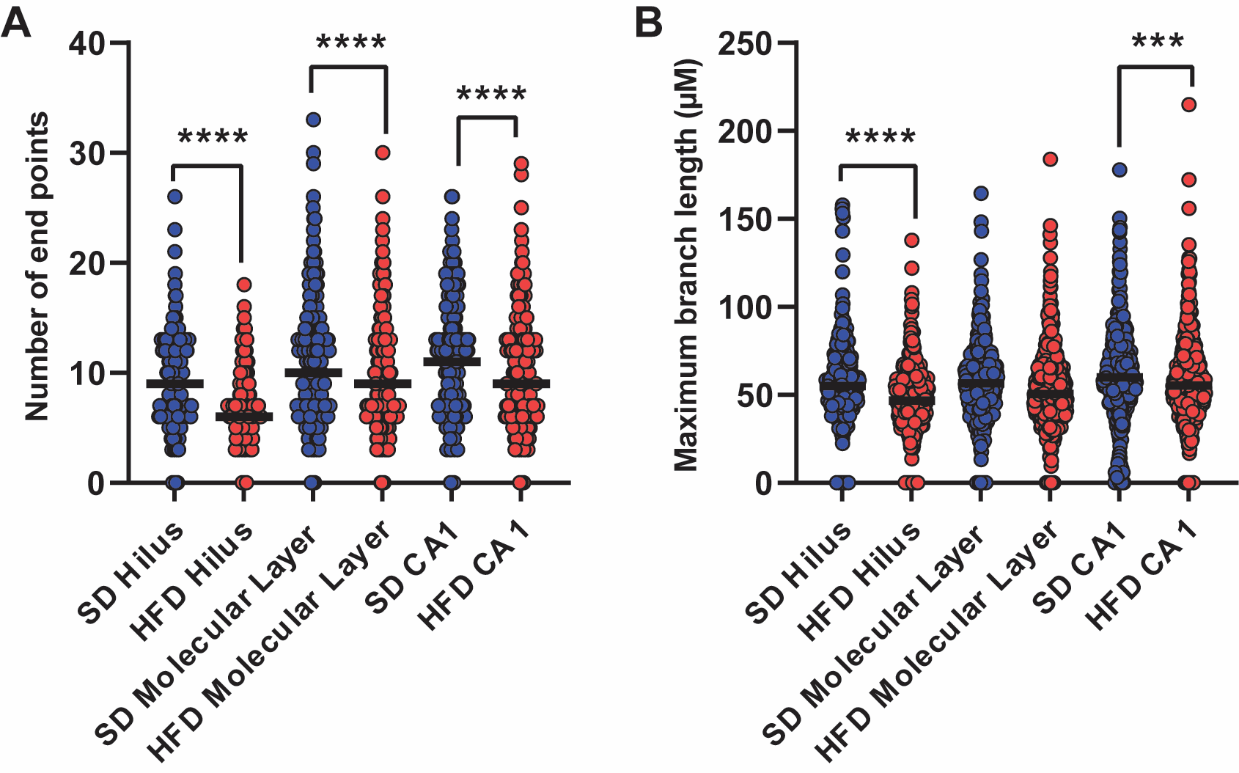


**Supplemental Fig 5.** Microglial morphology differences in cGAS -/- mice fed standard diet (SD) or high-fat diet (HFD) for 25 weeks per hippocampal region. Quantification of number of end points (A) and maximum branch length (B) in the hilus, molecular layer, and CA1 hippocampal regions. *n* = 8 SD, *n* = 9 HFD, ****p* < 0.001, *****p* < 0.0001, mixed model (SAS Proc Mixed) with number of cells per region set as a random effect. Data are presented as the mean ± s.e.m.


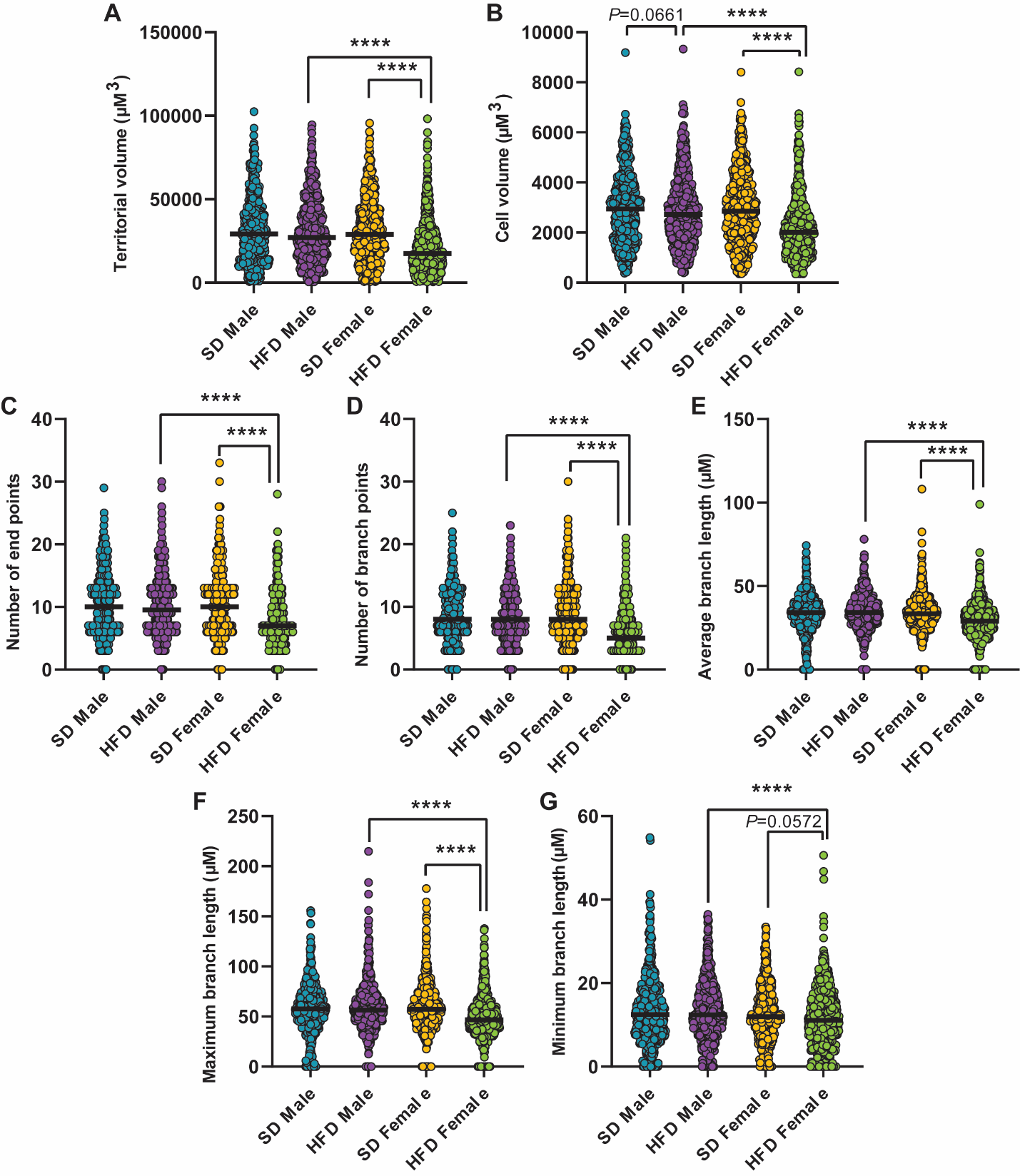


**Supplemental Fig 6.** Microglial morphology differences in male and female cGAS -/- mice fed standard diet (SD) or high-fat diet (HFD) for 25 weeks. Quantification of territorial volume (A), cell volume (B), number of end points (C), number of branch points (D), average branch length (E), maximum branch length (F), and minimum branch length (G) in the hippocampus. *n* = 4 male mice and *n* = 5 female mice/group, ****p* < 0.001, *****p* < 0.0001, mixed model (SAS Proc Mixed) with number of cells per region set as a random effect. Data are presented as the mean ± s.e.m.
