# Supplementary material for: Modeling the innate inflammatory cGAS/STING pathway: sexually dimorphic effects on microglia and cognition in obesity and prediabetes
Source: Front Cell Neurosci. 2023 May 3;17:1167688. doi: 10.3389/fncel.2023.1167688 (PMC10188944; doi:10.3389/fncel.2023.1167688)
Supplement: Supplementary file 2 [file Data_Sheet_2.docx]

**Supplemental Table 1.**

|  | **Male Dark** | **Male Light** | **Female Dark** | **Female Light** | **Female Dark** | **Female Light** | **Male Dark** | **Male Light** | **Male Fasted** | **Male Fed** |
| --- | --- | --- | --- | --- | --- | --- | --- | --- | --- | --- |
| **Strain** | cGAS-/- | cGAS-/- | cGAS-/- | cGAS-/- | C57BL/6J | C57BL/6J | C57BL/6J | C57BL/6J | Flox/flox controls for ANKO transgenics | Flox/flox controls for ANKO transgenics |
| **Manuscript** | Current | Current | Current | Current | Marvyn, 2016^1^ | Marvyn, 2016^1^ | Soofi, 2017^2^ | Soofi, 2017^2^ | Franczyk, 2021^3^ | Franczyk, 2021^3^ |
| **Timeline** | 24wk old | 24wk old | 24wk old | 24wk old | Unlisted | Unlisted | 20-24wk old | 20-24wk old | 13-26wk old | 13-26wk old |
| **EE** | 19.2 +/- 2.0 | 15.2 +/- 1.9 | 20.2 +/- 1.8 | 16.2 +/- 1.5 | N.A. | N.A. | 14 +/- 1.5 | 12 +/- 1 | +/- | +/- |
| **Activity** | 885 +/- 332 | 87 +/- 48 | 1113 +/- 508 | 160 +/- 113. | 400 +/- 100 | 300 +/- 75 | N.A. | N.A. | +/- | +/- |
| **RER** | 0.92 +/- 0.03 | 0.90 +/- 0.03 | 0.94 +/- 0.05 | 0.92 +/- 0.05 | 0.98 +/- 0.03 | 0.95 +/- 0.03 | N.A. | N.A. | 0.77 +/- 0.05 | 0.28 +/- 0.03 |
| **G.O.** | 3.7 +/- 0.7 | 2.8 +/- 0.7 | 4.3 +/- 1.1 | 3.2 +/- 0.9 | N.A. | N.A. | N.A. | N.A. | 2.25 +/- 0.5 | 8 +/- 1 |
| **F.O.** | 0.6 +/- 0.2 | 0.5 +/- 0.1 | 0.4 +/- 0.3 | 0.5 +/- 0.2 | N.A. | N.A. | N.A. | N.A. | 3.25 +/- 0.25 | -2.25 +/- 0.5 |
| EE=Energy expenditure (kcal/KgLBM/h); RER=Respiratory exchange ratio (VCO2/VO2); G.O.=Glucose oxidation (g/KgLBM/h); F.O.=Fat oxidation (g/KgLBM/h). Activity expressed as counts/h. Data from the cited literature interpolated from bar graphs presented in the manuscripts. | | | | | | | | | | |

**Supplemental Table 2**. Plasma cytokines (Saline vs LPS)

|  | **Male saline** | **Male LPS** | **Female saline** | **Female LPS** | **Male saline** | **Male LPS** | **Male saline** | **Male LPS** | **Male saline** | **Male LPS** |
| --- | --- | --- | --- | --- | --- | --- | --- | --- | --- | --- |
| **Strain** | cGAS-/- | cGAS-/- | cGAS-/- | cGAS-/- | C57BL/6J | C57BL/6J | C57BL/6 | C57BL/6 | C57BL/6 | C57BL/6 |
| **Manuscript** | Current | Current | Current | Current | Elzinga, 2022^4^ | Elzinga, 2022^4^ | Li, 2019^5^ | Li, 2019^5^ | Visitchanakun, 2021^6^ | Visitchanakun, 2021^6^ |
| **Timeline** | 43wk old | 43wk old | 43wk old | 43wk old | 4d diet, 6wk old | 4d diet, 6wk old | 8-10wk old | 8-10wk old | 8wk old | 8wk old |
| **Plasma IL-10** | 0 +/- 0 | 96.8 +/- 21.1 | 3.8 +/- 7.5 | 198.8 +/- 70.7 | N.A. | N.A. | N.A. | N.A. | 0 +/- 0 | 70000 +/- 2500 |
| **Plasma TNF-α** | 1 +/- 2 | 120.3 +/- 48.1 | 4.5 +/- 7.7 | 213.5 +/- 86.2 | 5.7 +/- 6.6 | 145.5 +/- 6.4 | 7 +/- 1 | 35 +/- 1.5 | 0 +/- 0 | 2100 +/- 1000 |
| **Plasma MCP-1** | 77.5 +/- 18.9 | 1125 +/- 0 | 155.3 +/- 83.1 | 1125 +/- 0 | 42.4 +/- 18.0 | 1125 +/- 0 | 15 +/- 3 | 85 +/- 3 | N.A. | N.A. |
| Li et al., LPS dose=10mg/kg IP, sacrificed 12h later; current manuscript and Elzinga et al. 2022, LPS dose = 500 μg/kg, sacrificed 4h later; Visitchanakun et al., LPS dose=10mg/kg IP, plasma collected 1, 3, and 6h post injection. Data expressed as pg/mL.; IP=intraperitoneal. Data from the cited literature interpolated from bar graphs presented in the manuscripts. | | | | | | | | | | |

**Supplemental Table 3.** Hippocampal cytokines (Saline vs LPS)

|  | **Male saline** | **Male LPS** | **Female saline** | **Female LPS** | **Male saline** | **Male LPS** | **Male saline** | **Male LPS** |
| --- | --- | --- | --- | --- | --- | --- | --- | --- |
| **Strain** | cGAS-/- | cGAS-/- | cGAS-/- | cGAS-/- | C57BL/6J | C57BL/6J | Swiss | Swiss |
| **Manuscript** | Current | Current | Current | Current | Zhao, 2019^7^ | Zhao, 2019^7^ | Fruhauf, 2015^8^ | Fruhauf, 2015^8^ |
| **Timeline** | 43wk old; | 43wk old | 43wk old | 43wk old | 12wk old | 12wk old | 12wk old | 12wk old |
| **Hippocampal IL-10** | 357.6 +/- 167.9 | 439.5 +/- 109.2 | 366.9 +/- 79.4 | 381.5 +/- 9.0 | N.A. | N.A. | 7.5 +/- 0.5 | 6.5 +/- 1 |
| **Hippocampal TNF-α** | 173.2 +/- 68.5 | 214.0 +/- 41.8 | 211.8 +/- 67.4 | 214.1 +/- 30.7 | 10 +/- 2 | 40 +/- 3 | 9 +/- 0.75 | 20 +/- 5 |
| **Hippocampal MCP-1** | 86.4 +/- 121.5 | 77.1 +/- 36.8 | 41.4 +/- 33.4 | 44.5 +/- 16.4 | N.A. | N.A. | +/- | +/- |
| **Hippocampal IL-6** | 92.4 +/- 68.3 | 117.1 +/- 42.9 | 101.8 +/- 35.4 | 101.1 +/- 3.2 | 10 +/- 1.5 | 32 +/- 3 | 6 +/- 0.5 | 18 +/- 3.5 |
| **Hippocampal IFN-γ** | 211.6 +/- 133.7 | 283.6 +/- 99.3 | 230.3 +/- 67.5 | 244.4 +/- 42.5 | N.A. | N.A. | 11 +/- 0.5 | 23 +/- 4 |
| Zhao et al., LPS dose=0.5mg/kg IP, sacrificed 24h later; Fruhauf et al., LPS dose=250 μg/kg, sacrificed 4h later; current manuscript, LPS dose = 500 μg/kg, sacrificed 4h later. Cytokine concentrations for the current manuscript are expressed as pg/mL per μg/μL protein. Data from the cited literature interpolated from bar graphs presented in the manuscripts. | | | | | | | | |

**Supplemental Table 4.** Metabolic Data (SD vs HFD)

|  | **SD Male** | **HFD Male** | **SD Female** | **HFD Female** | **SD Male** | **HFD Male** | **SD Female** | **HFD Female** |
| --- | --- | --- | --- | --- | --- | --- | --- | --- |
| **Strain** | cGAS-/- | cGAS-/- | cGAS-/- | cGAS-/- | C57BL/6J | C57BL/6J | C57BL/6J | C57BL/6J |
| **Manuscript** | Current | Current | Current | Current | Elzinga, 2021^9^ | Elzinga, 2021^9^ | Elzinga, 2021^9^ | Elzinga, 2021^9^ |
| **Timeline** | 25wk diet, 30wk old | 25wk diet, 30wk old | 25wk diet, 30wk old | 25wk diet, 30wk old | 19wk diet, 24wk old | 19wk diet, 24wk old | 19wk diet, 24wk old | 19wk diet, 24wk old |
| **Body Wt (g)** | 33.4 +/- 3.4 | 49.8 +/- 2.3 | 24.9 +/- 1.3 | 46.8 +/- 1.7 | 36.2 +/- 3.3 | 53.3 +/- 5.7 | 24.5 +/- 1.5 | 51.6 +/- 6.6 |
| **Insulin (ng/mL)** | 2.7 +/- 1.1 | 10.9 +/- 3.1 | 1.5 +/- 0.3 | 2.7 +/- 0.6 | 1.8 +/- 0.8 | 8.0 +/- 3.8 | 0.5 +/- 0.1 | 3.1 +/- 1.7 |
| **AUC** | 29274 +/- 5718 | 30848 +/- 4127 | 35693 +/- 9030 | 44802 +/- 13452 | 43769 +/- 7588 | 72956 +/- 13337 | 37572 +/- 4698 | 58343 +/- 7330 |
| AUC=Area under the curve. AUC of HFD males in the current study was significantly lower (*p*<0.05) compared to the AUC of HFD males from Elzinga et al., 2021. There were no other significant differences for body wt or plasma insulin concentrations. | | | | | | | | |

**Supplemental Table 5**. Plasma cytokines (SD vs HFD)

|  | **SD Male** | **HFD Male** | **SD Female** | **HFD Female** | **SD Male** | **HFD Male** | **SD Male** | **HFD Male** |
| --- | --- | --- | --- | --- | --- | --- | --- | --- |
| **Strain** | cGAS-/- | cGAS-/- | cGAS-/- | cGAS-/- | C57BL/6J | C57BL/6J | C57BL/6J | C57BL/6J |
| **Manuscript** | Current | Current | Current | Current | Henn, 2022^10^ | Henn, 2022^10^ | Elzinga, 2022^4^ | Elzinga, 2022^4^ |
| **Timeline** | 25wk diet, 30wk old | 25wk diet, 30wk old | 25wk diet, 30wk old | 25wk diet, 30wk old | 14wk diet, 20wk old | 14wk diet, 20wk old | 4d diet, 6wk old | 4d diet, 6wk old |
| **Plasma IL-10 (pg/mL)** | 3.7 +/- 4.0 | 19.0 +/- 30.8 | 7.3 +/- 1.5 | 14.5 +/- 18.4 | N.A. | N.A. | 0.0 +/- 0.0 | 11.2 +/- 18.8 |
| **Plasma TNF-α (pg/mL)** | 5.7 +/- 4.9 | 4.8 +/- 2.2 | 29.3 +/- 54.6 | 4.8 +/- 7.5 | 3.3 +/- 2.3 | 6.1 +/- 3.1 | 11.8 +/- 21.5 | 8.4 +/- 10.7 |
| **Plasma MCP-1 (pg/mL)** | 52.0 +/- 26.5 | 61.3 +/- 21.9 | 40.3 +/- 20.1 | 81.0 +/- 27.3 | 66.2 +/- 27.2 | 72.3 +/- 24.4 | 20.6 +/- 2.7 | 20.9 +/- 3.7 |
| **Plasma IL-6 (pg/mL)** | 5.3 +/- 4.0 | 3.0 +/- 4.1 | 0.5 +/- 1.0 | 1.3 +/- 2.5 | 1.0 +/- 1.7 | 0.2 +/- 0.7 | 1.6 +/- 2.6 | 2.3 +/- 4.2 |
| **Plasma IFN-γ (pg/mL)** | 3.3 +/- 4.2 | 1.3 +/- 2.5 | 1.3 +/- 1.0 | 0.5 +/- 1.0 | N.A. | N.A. | 4.8 +/- 3.4 | 11.9 +/- 17.6 |
| Young (6wk old) males from Elzinga et al., 2022 were significantly lower (*p*<0.05) in plasma MCP-1 compared to adult males (30wk old) in the current study. While not statistically different due to a high level of variation, HFD males from Elzinga et al., 2022 appeared to be higher in plasma IFN-γ and TNF-α compared to HFD cGAS-/- males in the current study. Data from the cited literature interpolated from bar graphs presented in the manuscripts | | | | | | | | |

**Supplemental Table 6**. Hippocampal cytokines (SD vs HFD)

|  | **SD Male** | **HFD Male** | **SD Female** | **HFD Female** | **SD Male** | **HFD Male** | **SD Male** | **HFD Male** |
| --- | --- | --- | --- | --- | --- | --- | --- | --- |
| **Strain** | cGAS-/- | cGAS-/- | cGAS-/- | cGAS-/- | C57BL/6J | C57BL/6J | C57BL/6J | C57BL/6J |
| **Manuscript** | Current | Current | Current | Current | Henn, 2022^10^ | Henn, 2022^10^ | Yang, 2019^11^ | Yang, 2019^11^ |
| **Timeline** | 25wk diet, 30wk old | 25wk diet, 30wk old | 25wk diet, 30wk old | 25wk diet, 30wk old | 14wk diet, 20wk old | 14wk diet, 20wk old | 6wk diet, 9wk old | 6wk diet, 9wk old |
| **Hippocampal IL-10** | 201.5 +/- 61.9 | 145.2 +/- 67.3 | 117.7 +/- 26.3 | 137.1 +/- 43.7 | 199.7 +/- 19.8 | 191.3 +/- 23.6 | N.A. | N.A. |
| **Hippocampal TNF-α** | 367.3 +/- 98.1 | 194.1 +/- 100.7 | 193.3 +/- 60.2 | 208.6 +/- 55.1 | **63.4 +/- 17.3** | **63.9 +/- 32.0** | 500 +/- 200 | 1000 +/- 200 |
| **Hippocampal MCP-1** | 37.4 +/- 9.3 | 22.9 +/- 10.2 | 21.4 +/- 5.1 | 21.6 +/- 5.2 | **11.8 +/- 3.4** | **11.3 +/- 5.5** | N.A. | N.A. |
| **Hippocampal IL-6** | 156.3 +/- 41.2 | 87.8 +/- 41.6 | 85.0 +/- 17.6 | 84.9 +/- 16.1 | **61.5 +/- 13.7** | 59.6 +/- 22.2 | 190 +/- 15 | 590 +/- 100 |
| **Hippocampal IFN-γ** | 248.4 +/- 59.1 | 167.0 +/- 52.3 | 164.1 +/- 21.2 | 167.7 +/- 22.8 | **67.0 +/- 14.2** | **66.7 +/- 21.2** | N.A. | N.A. |
| Cytokine concentrations for the current manuscript and for Henn et al., 2022 are expressed as pg/mL per μg/μL protein. Data from the cited literature interpolated from bar graphs presented in the manuscripts. | | | | | | | | |

**References**

1. Marvyn, P.M., et al., *Data on oxygen consumption rate, respiratory exchange ratio, and movement in C57BL/6J female mice on the third day of consuming a high-fat diet.* Data in brief, 2016. **7**: p. 472-475.

2. Soofi, A., et al., *The kielin/chordin-like protein (KCP) attenuates high-fat diet-induced obesity and metabolic syndrome in mice.* Journal of Biological Chemistry, 2017. **292**(22): p. 9051-9062.

3. Franczyk, M.P., et al., *Importance of adipose tissue NAD+ biology in regulating metabolic flexibility.* Endocrinology, 2021. **162**(3).

4. Elzinga, S.E., et al., *cGAS/STING and innate brain inflammation following acute high-fat feeding.* Front Immunol, 2022. **13**: p. 1012594.

5. Li, N., et al., *STING-IRF3 contributes to lipopolysaccharide-induced cardiac dysfunction, inflammation, apoptosis and pyroptosis by activating NLRP3.* Redox biology, 2019. **24**: p. 101215.

6. Visitchanakun, P., et al., *Interference on Cytosolic DNA Activation Attenuates Sepsis Severity: Experiments on Cyclic GMP–AMP Synthase (cGAS) Deficient Mice.* International Journal of Molecular Sciences, 2021. **22**(21): p. 11450.

7. Zhao, X., et al., *Behavioral, inflammatory and neurochemical disturbances in LPS and UCMS-induced mouse models of depression.* Behavioural brain research, 2019. **364**: p. 494-502.

8. Frühauf, P.K.S., et al., *Spermine reverses lipopolysaccharide-induced memory deficit in mice.* Journal of neuroinflammation, 2015. **12**(1): p. 1-11.

9. Elzinga, S.E., et al., *Sex differences in insulin resistance, but not peripheral neuropathy, in a diet-induced prediabetes mouse model.* Disease models & mechanisms, 2021. **14**(4): p. dmm048909.

10. Henn, R.E., et al., *Obesity-induced neuroinflammation and cognitive impairment in young adult versus middle-aged mice.* Immunity & Ageing, 2022. **19**(1): p. 67.

11. Yang, Y., et al., *Early-life high-fat diet-induced obesity programs hippocampal development and cognitive functions via regulation of gut commensal Akkermansia muciniphila.* Neuropsychopharmacology, 2019. **44**(12): p. 2054-2064.
